# A workflow for correlative *in situ* nanochip liquid cell transmission electron microscopy and atom probe tomography enabled by cryogenic plasma focused ion beam

**DOI:** 10.1039/d5nh00310e

**Published:** 2025-09-15

**Authors:** Neil Mulcahy, James O. Douglas, Syeda Ramin Jannat, Lukas Worch, Geri Topore, Baptiste Gault, Mary P. Ryan, Michele Shelly Conroy

**Affiliations:** a Department of Materials and London Centre for Nanotechnology, Imperial College London Exhibition Road London SW7 2AZ UK mconroy@imperial.ac.uk; b Max Planck Institute for Sustainable Materials Max-Planck-Str. 1 40237 Düsseldorf Germany

## Abstract

*Operando*/*in situ* liquid cell transmission electron microscopy (LCTEM) allows for real time imaging of dynamic nanoscale liquid-based processes. However, due to the thick liquid cell of traditional LCTEM holders and thus scattering of the electron beam passing through the cell, the achievable spatial and chemical resolution is limited. Cryogenic atom probe tomography (cryo-APT) overcomes these limitations by offering (near-)atomic scale compositional analysis of frozen liquid–solid interfaces. However, APT provides limited structural analysis and has no capacity for dynamic or *operando* liquid cell studies. This work presents a novel workflow for site-specific cryo-APT sample preparation of liquid–solid interfaces from *in situ* electrochemical LCTEM micro-electro-mechanical systems (MEMS) chips. Using a cryogenic inert gas transfer suitcase and a cryogenic plasma-focused ion beam (PFIB), a MEMs nanochip containing a Li electrolyte from an electrochemistry LCTEM holder was successfully frozen, transferred to the cryo stage of a PFIB and prepared into APT needle samples containing the electrolyte–electrode interface at cryogenic temperatures, followed by cryogenic transfer to an atom probe for nanoscale compositional analysis. This correlative approach enables both dynamic nanoscale imaging and near atomic scale compositional analysis of air sensitive and reactive liquid–solid interfaces. This method enables reliable and reproducible APT sample preparation of these frozen interfaces from MEMs based nanochips and can hence be used across materials systems and energy-conversion or storage devices.

## Abstract 2

*Operando*/*in situ* liquid cell transmission electron microscopy (LCTEM) allows for real time imaging of dynamic nanoscale liquid-based processes.

## Graphical abstract

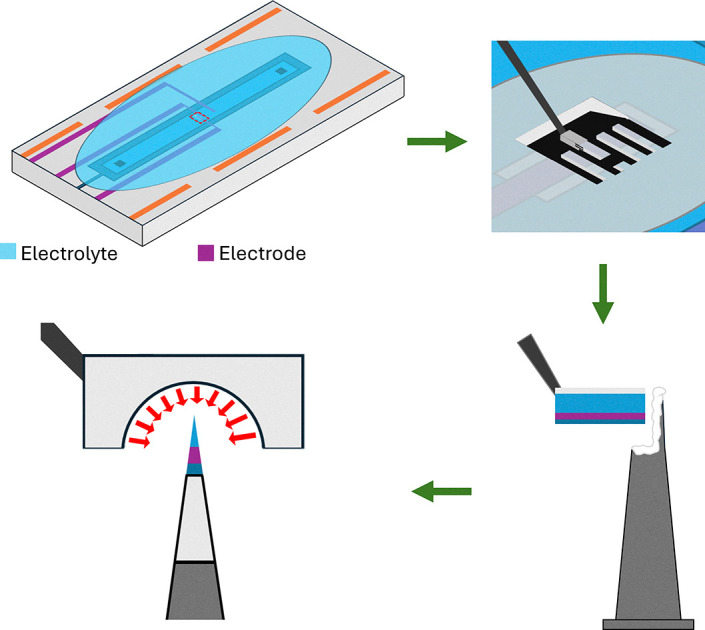

**New concepts**

How can we correlate dynamic behaviour in liquids with atomic-scale insights at liquid–solid interfaces, particularly when such interfaces must be studied under high vacuum? This remains a fundamental challenge in characterising soft, beam-sensitive materials, where key nanoscale processes—such as ion transport and interfacial reactions—occur in transient or buried environments. In this work, we present a correlative methodology that combines electrochemistry liquid cell transmission electron microscopy (E-LCTEM) with cryogenic atom probe tomography (cryo-APT) to overcome this barrier. Following a “see, freeze, and resolve” approach, E-LCTEM enables real-time imaging of nanoscale dynamics in liquid media, while cryo-APT provides three-dimensional, near-atomic resolution chemical mapping of the vitrified system under ultra-high vacuum. We demonstrate this workflow using lithium-based electrolytes, capturing liquid-phase transformations and subsequently resolving atomic-scale interfacial chemistry at electrode–electrolyte boundaries. This dual technique bridges the long-standing divide between *in situ* visualisation and vacuum-compatible, high-resolution chemical characterisation. Our findings offer a broadly applicable strategy for investigating reactive liquid–solid interfaces, opening new avenues in electrochemical systems, battery materials, and interfacial nanoscience. By correlating structural evolution with chemical resolution, this approach provides a new conceptual framework for studying soft and dynamic materials at the atomic scale.

## Introduction

Transmission electron microscopy (TEM), electron spectroscopy and diffraction are valuable tools in materials science research for investigating the structural, chemical and compositional properties of a variety of different material systems and processes.^1^ While traditional TEM techniques can provide up to atomic scale insights into a variety of properties,^2,3^ the majority of characterization is done in a static, often vacuum state. In reality, understanding material behavior often requires analyzing materials and their interfaces under dynamic, real-world conditions that reflect their functional use.^4–6^ Typically, TEM experiments require high vacuum conditions,^7^ thus limiting the analysis of samples in liquid or gaseous environments.^8^ One way the TEM research community has overcome these vacuum constraints is the use of specialized cells that encapsulate samples between thin, electron-transparent viewing windows.^9^ The introduction of micro-electro-mechanical systems (MEMS)-based technology has further advanced *in situ* TEM by facilitating not only the ability to encapsulate the sample in a gas or liquid environment, but also to directly heat or bias the samples on nanochips. Combined with new *in situ* holder designs, MEMS-based TEM allows for the precise control of the sample environment and applied stimulus while conducting imaging, diffraction and/or spectroscopy.^10–13^ One can now probe an extensive list of material phase transformations such as degradation and growth mechanisms in battery materials^14–16^ and catalysts,^17,18^ and other functional materials in real time at high spatial resolutions.

**Fig. 1:**
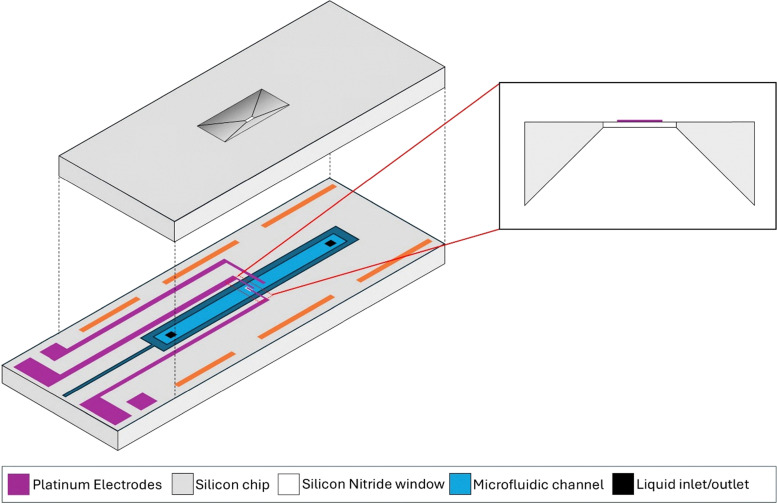
A schematic illustration of a liquid cell electrochemical nanocell, composed of a top and bottom MEMS-based silicon nanochip. The SiN_*x*_ membrane window with printed electrode is highlighted.

One area which has grown in popularity over the last decade due to this MEMS nanochip revolution, is the field of *in situ*/*operando* liquid cell TEM (LCTEM). LCTEM enables real-time observation of both static and dynamic processes in solution-phase or immersed materials, with high temporal and spatial resolutions.^8^ This is achieved through the use of a closed cell environment utilising MEMS chip technology. The liquid is allowed to flow through the system of interest using external liquid supply systems or syringe pumps^19,20^ while being viewed through electron transparent silicon nitride membranes (SiN_*x*_) on both the bottom and top nanochips. Novel MEMS chip designs have enabled electrical contacts and heating elements to be fitted to the bottom nanochip allowing *in situ*/*operando* electrical biasing and heating experiments within liquid environments.^14,21^ This type of design is also used for *in situ* gas closed cell experiments that require heating or biasing.^22–26^ A schematic illustration of a representative nanocell is shown in Fig. 1. This type of experimental set up has provided insights into a number of dynamic nanoscale phenomena such as nanoparticle synthesis and growth,^27,28^ corrosion^29,30^ and electrochemical phenomena such as dendrite growth^14,31–33^ and solid–electrolyte interphase (SEI) formation.^15,34^

While the usefulness of this microscopy technique for investigating liquid processes at the nanoscale has been detailed in numerous reports, the technique still presents key problems particularly with respect to reduced spatial resolution^35^ and electron beam induced effects.^36,37^ Reduced spatial resolution in LCTEM in comparison to traditional TEM/(S)TEM largely occurs due to increased electron scattering as a result of increased sample volumes. The overall cell thickness typically comprises of the liquid, the electron transparent membrane windows and the sample of interest. Often this can lead to an overall cell thickness in excess of 100 nm, making it difficult for electrons to be transmitted through the cell. This means imaging and spectroscopy techniques such as electron diffraction or electron energy loss spectroscopy (EELS) are either at significantly lower resolutions or cannot be performed at all. LCTEM studies are therefore typically only capable of generating dynamic nanoscale imaging and the only available way of determining any compositional changes is through the generated contrast, be it from phase- or amplitude-contrast. While various solutions have been proposed and implemented, such as altering cell designs involving 2D materials^15,38–40^ and utilising dose limitation techniques, many of these solutions remain novel and challenging to apply to commercially available liquid cell systems and nanochips. Initial publications combining closed liquid cell with biasing have allowed for near atomic resolution, however liquid flow is not possible in these set-ups,^41,42^ meaning solution concentrations are not well controlled over time.

The ability to provide both dynamic nanoscale imaging and nanoscale compositional analysis within liquid environments would be invaluable for problems affecting numerous material systems, including within battery research. However, any such correlative approach would require the liquid environment to remain in its state of interest. In recent years, cryogenic microscopy techniques such as cryogenic TEM/STEM and cryogenic atom probe tomography (cryo-APT)^43–47^ have been used to provide near atomic scale compositional insights into various liquids and liquid–solid interfaces.^46,48^ Such cryogenic analysis has been realised through advances in site specific specimen preparation workflows by using cryogenic-focused ion beam/scanning electron microscopy (FIB/SEM).^49–53^ While cryo-APT uniquely combines chemical sensitive, high resolution 3D compositional mapping, it has no capacity for dynamic or *operando* studies and only provides snapshots of the evolution of a particular system, with limited crystallographic or structural analysis. This makes information provided by *in situ*/*operando* LCTEM and cryo APT extremely complementary to one another.

In this work, we present a novel correlative workflow which provides site-specific cryo-APT specimen preparation of a liquid solid-interface from a liquid cell electrochemical MEMs-based nanochip. By integrating cryogenic inert gas transfer technology and a cryogenic plasma-FIB/SEM (PFIB/SEM), we have successfully frozen a MEMs chip covered in a lithium electrolyte from a commercial LCTEM holder, transferred the frozen interface to the cryo stage of a PFIB/SEM and prepared reliable and reproducible cryo-APT specimens from the frozen liquid–solid interface. The created cryogenic samples were transferred directly to the analysis chamber of an atom probe instrument under cryogenic conditions. We captured the 3D nanoscale compositional analysis of the frozen liquid–solid interface from the MEMs nanochip.

## Instrumentation and materials

### Liquid cell transmission electron microscopy system and MEMs chips

A Thermofisher Scientific (Waltham, Massachusetts, United states) Spectra 300 (S)TEM at 300 kV accelerating voltage was used for all STEM measurements. This instrument is probe corrected and fitted with an ultra-high-resolution X-FEG Ulti-monochromator. The measured screen current during imaging was 47 pA, which equates to an electron dose of 1.22 × 10^3^ e Å^−2^. High angle annular dark field (HAADF) imaging was used for all (S)TEM images shown.

The Stream system, including the *in situ* liquid TEM holder and the pressure based liquid supply system (LSS), supplied by DENSsolutions B.V. (Delft, The Netherlands) was used for all work involving LCTEM. A nanocell is comprised of a top and bottom silicon wafer chip. The MEMS based bottom nanochip contains a three-electrode set up, with a platinum (Pt) reference, counter and working electrode. The working electrode is deposited on a 50 nm electron transparent SiN_*x*_ membrane window. This window has dimensions of approximately 20 μm × 200 μm.^21^ The top chip contains an identical electron transparent membrane window, allowing for viewing of the working electrode within the microscope. Prior to assembly, the bottom nanochip is plasma cleaned in Ar–O for approximately 3 minutes. This will make the nanochip hydrophilic, which is advantageous when trying to freeze the sample to avoid large droplets of liquid. A commercial lithium electrolyte, LiPF_6_ in ethylene carbonate/diethyl carbonate (EC/DEC), supplied by Merck Life Science UK Ltd (Dorset, United Kingdom), was flowed through an assembled cell within the TEM. The flow was controlled by the LSS in combination with Impulse, a commercially available software from DENSsolutions B.V. (Delft, The Netherlands). Within the software an inlet and outlet pressure of 2000 and −950 mbar respectively were selected, resulting in a flow rate of ≈8 μL min^−1^.

### Vacuum cryo transfer module and glovebox

Samples could be transferred under vacuum/inert conditions and also at constant cryogenic temperatures between instruments using a vacuum cryo transfer module (VCTM), supplied by Ferrovac GmbH (Zürich, Switzerland). The module is equipped with a small ion pump with a non-evaporable getter cartridge, allowing the module to maintain a pressure of 10^−10^ mbar. Cryogenic temperatures can be maintained within the module using a dewar of liquid nitrogen (LN_2_). The module can accept industry standard pucks and cryo pucks supplied by CAMECA Inc. (Gennevilliers, France). The system contains a 500 mm wobblestick with a PEEK-insulated puck manipulator allowing pucks to be picked up or released.^51^

All cryogenic sample preparation was conducted using an inert glovebox, supplied by Sylatech Ltd (York, United Kingdom). This glovebox contains an inert nitrogen atmosphere with typical oxygen content and humidity both below 5 ppm during operation. It has a large load lock, allowing for large samples or even entire TEM holders to be inserted directly into the glovebox chamber. LN_2_ can be pumped directly into a bath within the glovebox using a large pressured dewar external to the system, Apollo 50, supplied by Cryotherm Inc. (Kirchen (Sieg), Germany). Samples can be plunge frozen within the glovebox and transferred to a VCTM using a combination of the LN_2_ bath and a cooled “elevator” contained within a loadlock chamber capable of being pumped to vacuum. This loadlock chamber is connected to a Ferroloader docking station, supplied by Ferrovac GmbH (Zürich, Switzerland). Frozen samples can be picked up directly from the elevator loadlock chamber using a wobblestick within the VCTM and pulled into the module, maintaining the sample under vacuum and at constant cryogenic temperatures throughout the entire process.

### Cryogenic plasma focused ion beam/scanning electron microscope

A Helios Hydra CX (5CX) plasma FIB from Thermo Fisher Scientific (Waltham, Massachusetts, United states) fitted with an Easylift tungsten (W) cryo-micromanipulator and an Aquilos cryo-stage was used for all FIB/SEM work shown. Through the circulation of gaseous nitrogen passing through a heat exchanger within a large external dewar of LN_2_, the stage and micromanipulator can be cooled to approximately 90 K. To achieve this base temperature, a nitrogen gas flow of 180 mg s^−1^ was maintained. The temperature of both the stage and micromanipulator are controlled using a temperature control unit, Model 335 cryogenic temperature controller supplied by LakeShore Cryotronics Inc (Westerville, Ohio, United States) in combination with heaters built into the stage. The system is also equipped with a Ferroloader docking station. This allows a precooled VCTM to be docked to the side of the instrument and cryogenic samples under vacuum can be inserted directly to the cryo stage of the FIB from the module without heating up.^51^

For the purpose of this work, a “Dual-puck” holder stage baseplate supplied by Oxford Atomic (Oxford, United Kingdom) was used. This allows for two industry standard cryo pucks supplied by CAMECA Instrument Inc. (Madison, WI, USA), to be inserted to the cryo stage at once. The typical configuration used for these experiments was one puck containing the frozen liquid cell nanochip, while the other puck would contain a pre-prepared silicon (Si) microarray coupon. The Si microarray coupon was prepared at room temperature prior to any cryogenic work. This involved pre-cutting the posts at 0°, while also precoating the posts and micromanipulator in SEMGlu^TM^, supplied by Kleindiek Nanotechnik GmbH (Reutlingen, Germany).^54,55^ This pre-preparation procedure is reported in detail by Mulcahy *et al.*^56^ The FIB column is set at 52° with respect to the SEM column. Any FIB work shown used Xenon (Xe) plasma.

### Atom probe tomography

A Local Electrode Atom Probe (LEAP) 5000 XR supplied by CAMECA Instruments Inc. (Madison, WI, USA) was used for all atom probe analysis shown. This instrument is equipped with a reflectron system and a Ferroloader docking system. This means a VCTM can be docked directly to the atom probe and a specimen can be inserted onto the cryo stage located in the atom probes analysis chamber through the use of a “piggyback” puck while being maintained under vacuum and at cryogenic temperatures. The sample was analysed using laser pulsing analysis (40–80 pJ, 80–240 kHz, 1 ion per 500 pulses on average, 25k base temperature). 3D reconstructions and atom probe data analysis were completed using AP suite 6.3, a commercially available software from CAMECA Instruments Inc. (Madison, WI, USA).

## Results and discussion

### Liquid cell TEM

**Fig. 2:**
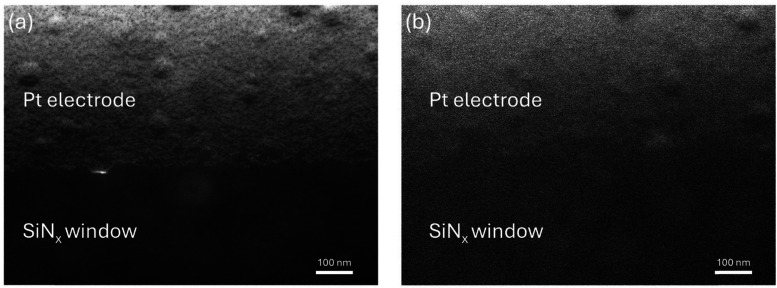
HAADF STEM images of a Pt working electrode during a LCTEM experiment: (a) prior to electrolyte flow, and (b) during the introduction of the Li electrolyte (LiPF_6_ in EC/DEC) into the nanocell. The reduction in resolution observed in (b) is due to increased electron beam scattering from the liquid within the system.

Fig. 2 shows HAADF STEM images of (a) an electrode prior to any liquid flow and (b) after Li electrolyte has flown into a nanocell under open circuit conditions (no bias applied). The change in imaging resolution and quality is apparent following liquid flow. This decreased resolution is largely occurring as a result of increased electron beam scattering due to the thickness of the liquid within the nanocell. The liquid was flowed through the system for ten minutes to allow a reactive liquid–solid interface to form as a model system to showcase this correlative workflow.

### Freezing process

**Fig. 3:**
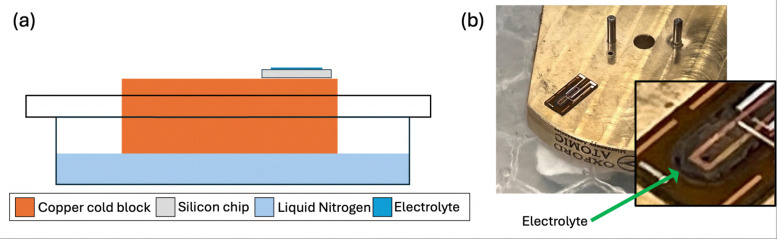
(a) Schematic illustrating the freezing process, showing a copper block submerged in LN_2_ with an electrolyte-covered nanochip placed on top. (b) Photograph of the freezing process, displaying a noticeable layer of frozen electrolyte on the liquid cell nanochip.

At the end of the LCTEM experiment, both inlet and outlet valves on the holder were closed, ensuring liquid remained within the system and free from direct air contact, and all connections from the holder to the LSS were completely removed. Simultaneously, a copper block (in this instance the “Dual-puck” holder stage baseplate supplied by Oxford Atomic) was placed in a small bath of LN_2_ and allowed to cool to LN_2_ temperatures (≈77.15 K), making it act as a “cold block”. The LCTEM holder was removed completely from the TEM and disassembled in air. Upon disassembling the bottom nanochip contained a noticeable layer of liquid electrolyte over the electrodes/SiN_*x*_ membrane window. This bottom chip is subsequently placed on the cold block to rapidly cool the system to LN_2_ temperatures, preserving the liquid–solid interface frozen in its state of interest. Following noticeable freezing of the liquid on the chip, the entire chip was then completely submerged in LN_2_ to protect it from frost build up. Fig. 3 shows an illustration and photograph of this freezing process, highlighting the setup used. The process of disassembling the cell and placing it on the cold block would routinely take less than 10 seconds.

While Li-containing electrolytes, electrodes and Li decomposition products are air- and moisture-sensitive,^57–59^ it was deemed that speed was a more important factor when freezing the bottom nanochip, and that immediately disassembling the nanocell in air rather than transferring to the inert nitrogen glovebox and subsequent freezing would lead to better preservation of any surface products (such as SEI components), and any degradation of the region of interest would be protected to some extent from oxygen by the volume of electrolyte covering it. Further to this, previous experiments by the author had shown that directly plunge freezing the nanochip in LN_2_ resulted in the loss of decomposition products. Slower cooling on a cold block was found to work better for maintaining the region of interest of the sample. While this method could lead to increased frost build up on the chip during cooling the goal was to ensure reduced decomposition of the region of interest (ROI).

### Transfer process

**Fig. 4:**
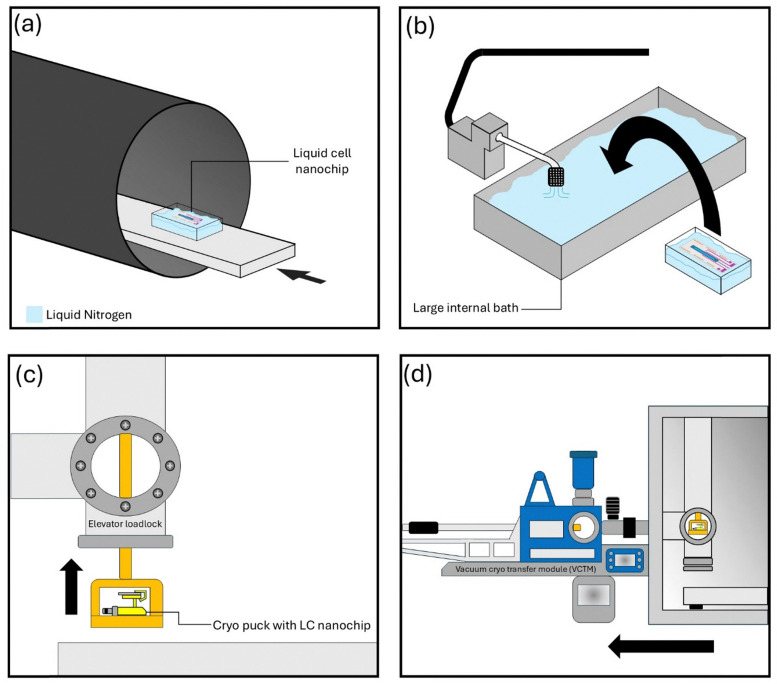
Schematics illustrating the transfer process for moving a frozen MEMS nanochip to the cryo stage of a PFIB/SEM through the use of an inert glovebox and a VCTM. (a) The frozen nanochip is inserted into the glovebox through a small side antechamber using a small volume of LN_2_. (b) The frozen nanochip is moved from this small volume to a larger LN_2_ bath within the glovebox, maintaining the system's state of interest. (c) The frozen nanochip is placed into a cryo clip on a cryo puck and inserted into the elevator within the glovebox. This elevator can be raised into a loadlock chamber and pumped to UHV. (d) Under UHV, the frozen nanochip is transferred directly to a precooled VCTM.

The next step of the workflow involves transferring the now frozen liquid–solid interface on the MEMs nanochip to the cryo stage of a PFIB/SEM. This is achieved through the use of an inert nitrogen glovebox and a VCTM. Fig. 4 presents schematics of the step-by-step procedure involved in transferring a frozen sample through the glovebox, into a VCTM and subsequently to the cryo PFIB/SEM. Frozen nanochips can be introduced into the glovebox through a side antechamber using a small volume of LN_2_, illustrated in Fig. 4(a). This process will evaporate a small amount of LN_2_ during the pumping and purging of the antechamber. However, it was found to be sufficient to maintain the sample in its frozen state. Within the glovebox a larger bath can be filled with LN_2_ from an external pressurised dewar. The frozen nanochip is inserted into this larger bath, ensuring the ROI remains frozen. This process is shown in Fig. 4(b). From here the frozen nanochip is placed into a cryo clip on a cryo puck, which is compatible with the VCTM, the stage baseplate of the cryo PFIB/SEM, and the APT instrument. A loadlock within the glovebox can be cooled using the external pressurised dewar, and the frozen nanochip on the cryo puck can be inserted into this loadlock through the use of an elevator, as seen in Fig. 4(c). The elevator loadlock can be maintained at approximately 115 K. This loadlock can be closed and subsequently pumped to ultra-high vacuum (UHV). The vacuum level and temperature within this loadlock are sufficient to prevent frost build up, while maintaining the sample in its frozen state. From here the sample can be pulled into a precooled VCTM, which once filled with LN_2_ can maintain a temperature of approximately 90 K, as shown in Fig. 4(d). The VCTM can be detached from the glovebox, loaded onto the PFIB/SEM using a Ferroloader docking station, and the frozen sample can be inserted directly to the cryo stage of the PFIB/SEM, without heating up or building up frost.

### Cryogenic FIB/SEM preparation

**Fig. 5:**
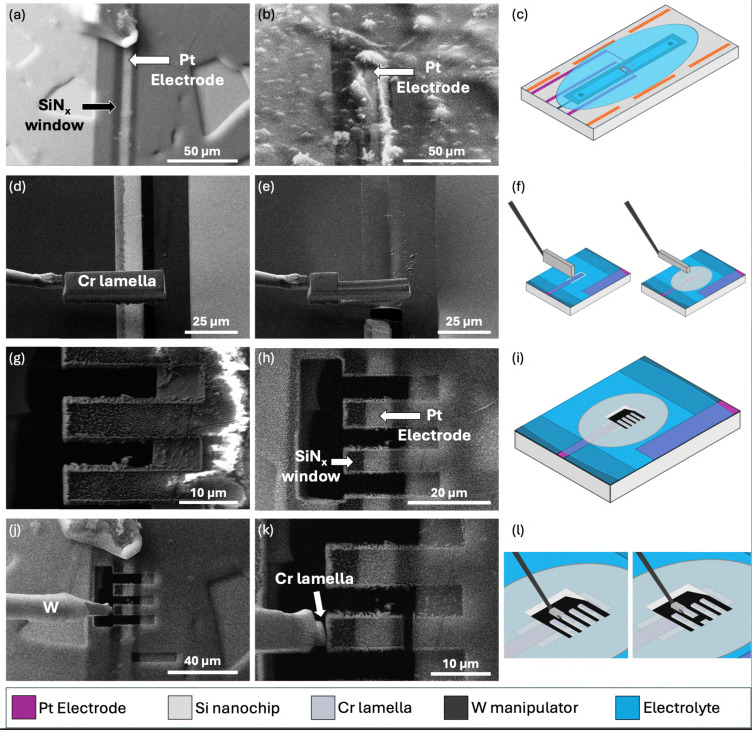
SEM micrographs and schematics illustrating the ROI identification, Cr protective layer coating, milling and lift out process. (a) and (b) Show two examples of a Pt electrode covered in electrolyte, while (c) highlights the specific region of the MEMS nanochip being observed. (d) and (e) Demonstrate the application of a Cr protective layer over the ROI, with (f) providing a schematic representation of this process. (g) and (h) Show the ROI milled into lift-out bars; (g) was acquired at 5 kV and (h) at 25 kV, illustrating the increased visibility of the electrode at higher accelerating voltages. (i) Presents a schematic of the lift-out bars in relation to the full MEMS nanochip. (j) Shows the attachment of a lift-out bar to the micromanipulator using redeposition welding with a Cr lamella, and in (k) the sample is milled free and lifted out. (l) Provides a schematic overview of the full lift-out process.

The aim for this part of the workflow is to create a specimen appropriate for APT analysis from the frozen liquid–solid interface involving the Pt electrode and Li electrolyte. APT specimen requirements involve creating a needle-like geometry with a diameter of approximately 100 nm or below at the apex.^48^ The first step for this process involves identifying the electrode–electrolyte interface ROI using SEM imaging. Fig. 5(a) and (b) show two separate examples of a Pt electrode covered in electrolyte. The uniformity of the electrolyte on the electrode can vary substantially between experiments, often making it difficult to identify the exact location of the ROI. This non-uniformity can be attributed to various parameters such as the flow rate of liquid during the *in situ* experiment, any inherent heterogeneities present in the samples, the freezing process, and whether the nanochip has been plasma cleaned prior to insertion into the liquid cell holder. It is recommended to use a higher kV electron beam (20–25 kV) to directly see the electrodes through the electrolyte. If even at higher kVs the electrodes cannot be identified due to the thickness of the electrolyte, milling around the ROI may be required. Due to the thin nature of the SiN_*x*_ membrane window and electrode directly milling will quickly leave a hole which is easy to identify in comparison to milling a bulk part of the nanochip. In this study, accurately identifying the ROI observed during the LCTEM experiment is challenging, however we can identify the electrode region by measuring where on the length of the electrode the ROI was in the TEM and again in the PFIB. Of course, due to the absence of distinct or persistent features it is vital the measurement of ROI region along the length of the electrode is as accurate as possible. In addition for experiments involving electrochemical biasing or nanoparticle growth, the emergence of identifiable morphological changes often facilitates more reliable correlation between LCTEM and subsequent FIB/SEM preparation.

Once the ROI has been identified it is necessary to add a protection layer prior to any further milling to protect the interface from damage from the ion beam. At room temperature this protection layer is typically achieved through the use of a decomposed organometallic gas injected *via* a gas injection system (GIS), forming site specific layers of various metals such as Pt or W, or other species such as C.^60^ However, at cryogenic temperatures this site-specific deposition is more challenging to control as the precursor gas will condensate over mm^2^ sized areas of the cold sample surface,^61,62^ ultimately losing the site specificity of the process. This can make it exceptionally challenging to identify the ROI. While possibilities of controlling this process have been detailed by Parmenters *et al.*,^63^ a more reliable site-specific method is required. As described by Schwarz *et al.*^52^ and for flat surfaces by Woods *et al.*,^50^ site specific *in situ* deposition of a metallic layer can be achieved by rastering the ion beam over a lamella attached to the micromanipulator that is in close proximity to the sample's surface. This process is demonstrated in Fig. 5(d) and (e), where the Xe ion beam (30 kV, 1 nA) is being rastered over the surface of a Cr lamella, coating the surface of the electrolyte–electrode in a thin site-specific protective layer of Cr.

Ensuring a sufficient protective layer is covering the ROI, the FIB stage is tilted to 52° and using the Xe plasma beam (30 kV, 1–4 nA) the electrodes of interest were milled into lift out bars, taking care not to damage the ROI at the apex of the electrodes. An example of this can be seen in Fig. 5(g) and (h). Due to the thin nature of the SiN_*x*_ membrane window and electrode an undercut is not performed, and attempting to create an undercut could potentially result in damaging the ROI. Again, due to the difficulties involved in using the GIS at cryogenic temperatures the created lift out bars could be lifted out using a preprepared Cr lamella using redeposition welding (Xe, 30 kV, 30 pA), Fig. 5(j) and (k), as described by Douglas *et al.*^49^ and Woods *et al.*^50^ Schematics of the electrolyte–electrode interface identification, Cr coating, milling and lift out procedure can be seen in Fig. 5(c), (f), (i) and (l).

**Fig. 6:**
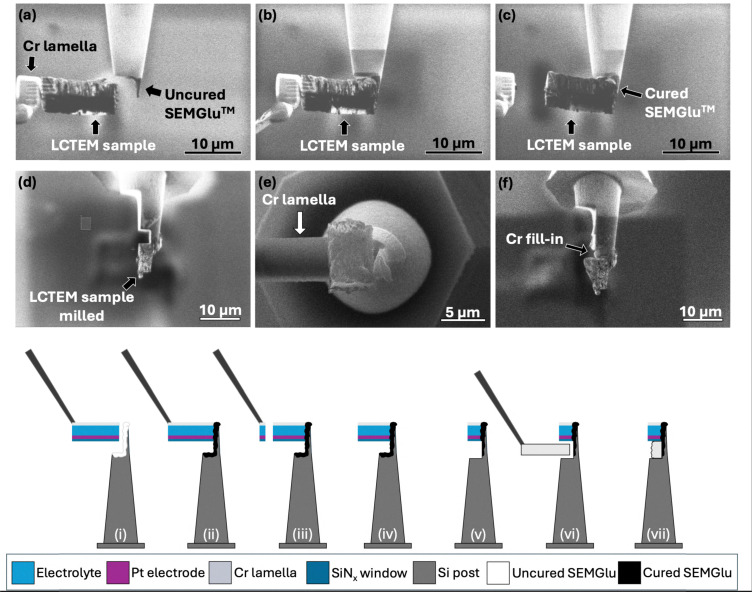
(a)–(c) SEM micrographs showing the attachment of the lift-out bar to a Si microarray post using SEMGlu™. Note the change in contrast of the SEMGlu™ between (a) and (c), indicating curing of the adhesive. (d) An SEM micrograph showing the sample milled to match the dimensions of the post with the interface modified between the lift-out bar and post to enable a more secure connection. (e) An SEM micrograph showing the sample being filled in *via* a Cr lamella. (f) An SEM micrograph showing an example of the same sample after it has been filled in using Cr redeposition. This entire procedure is schematically illustrated in (i)–(vii).

Due to the difficulty with creating an undercut when lifting out the electrode–electrolyte interface it can be challenging to securely attach a sample to a Si microarray post at cryogenic temperatures using redeposition welding alone. This is due to the beam sensitive nature of the sample, and the lack of good surface contact between the post and the sample. As shown in Fig. 6(a)–(c), the lift out bar can be brought in contact with frozen SEMGlu^TM^ on a preprepared Si microarray post. This glue can be cured using the ion beam (Xe, 30 kV, 30 pA), and the sample will remain securely stuck to the post once milled free from the micromanipulator without further assistance.^56^ Note the changing contrast of the glue once cured. Following this the sample is milled to fit the dimensions of the Si post, as seen in Fig. 6(d), and the connection between the post and the sample is also milled to allow for a more secure contact to be created using redeposition from a Cr lamella, as described Douglas *et al.*^49^ and shown in Fig. 6(e). This is done to ensure the sample is mechanically stable and electrically conductive throughout its entire volume. An example of a filled in sample can be seen in Fig. 6(f). This entire procedure is schematically shown step by step in Fig. 6(i)–(vii).

**Fig. 7:**
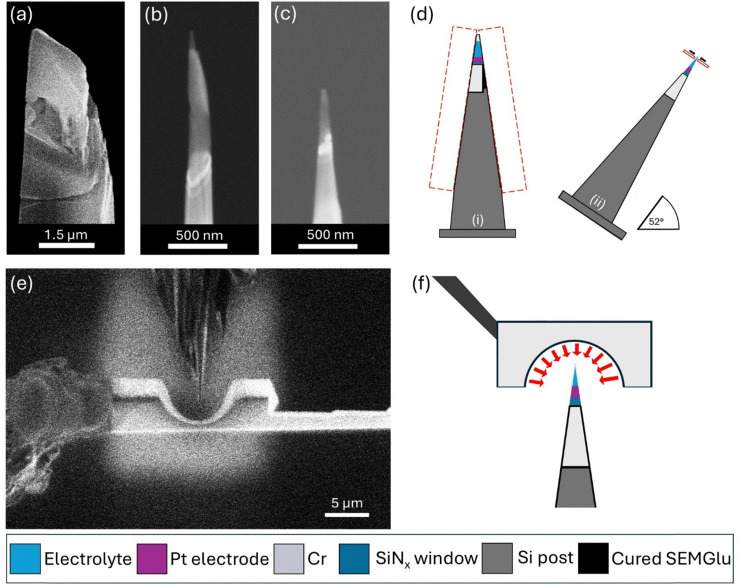
SEM micrographs showing a sample after (a) rough milling at 0° stage tilt, (b) fine milling at 52° stage tilt, and (c) following a low kV ion beam polish. (d) Showcases a schematic of both the rough and fine milling procedures. (e) An SEM micrograph of the coating process with (f) representing this schematically.

At this point the sample is ready to be milled into a needle shape with the aim of creating a specimen with a diameter of roughly 100 nm at the apex. Initially a rough needle shape was created at shallower and shallower shank angles at 0° stage tilt and rotating periodically using a series of rectangular box milling patterns with the ion beam (Xe, 30 kV, 1–4 nA) until a rough diameter of around 1 μm was achieved. The sample is then rotated to 52° stage tilt and again using a series of rectangular box milling patterns with the ion beam (Xe, 30 kV, 10 pA–0.3 nA) the sample was thinned to be roughly 200 nm in diameter. The difference in sample diameter is highlighted in Fig. 7(a) and (b). In this work, box milling patterns were used instead of conventional annular milling due to the lower resolution and broader beam profile associated with Xe ion sources.^64^ Annular milling at small diameters was found to be too aggressive, often resulting in damage to the delicate liquid–solid interface. A final low kV Xenon plasma beam polish was used to create the final needle shape which can be seen in Fig. 7(c). Schematics of both milling processes are shown in (d). Varying layers are evident in this final needle including electrolyte, Pt electrode and Cr weld indicating that an interface has been captured. As a final preparation step the needle was coated in Cr using redeposition from the ion beam (Xe, 30 pA, 30 kV) on all four sides for 20 s. This process has been described in detail in a number of reports.^52,65,66^ Metallic coatings have been shown to offer an effective shield against *in situ* delithiation effects that can occur during APT analysis due to the applied electrostatic fields.^65^ The coating has also been shown to provide increased mechanical support and superior pathways for heat dissipation. An SEM micrograph of this process and a schematic can be seen in Fig. 7(e) and (f). Created frozen needles containing the electrolyte–electrode interface could then be transferred directly to the analysis chamber of the APT instrument using a pre-cooled VCTM, maintaining the samples in their frozen state and under vacuum throughout the entire process.

### Cryogenic APT

**Fig. 8:**
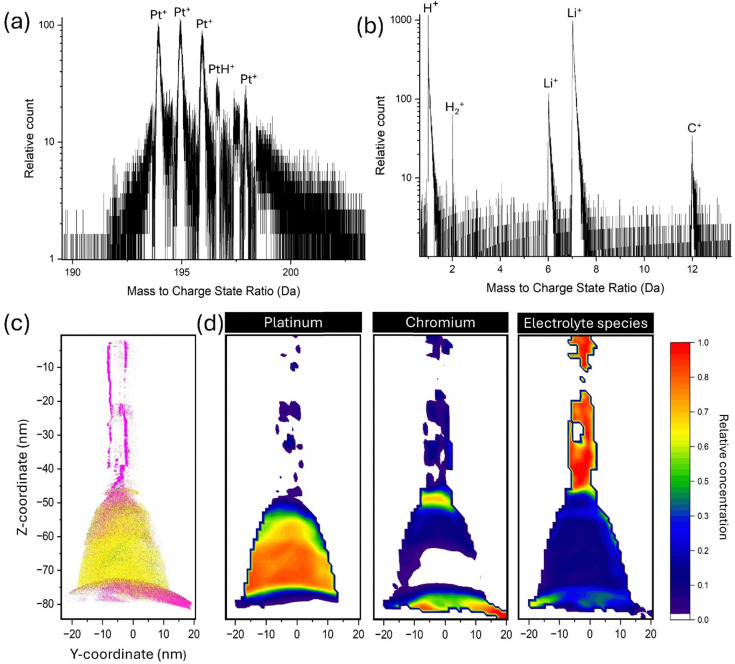
(a) Displays a portion of the generated mass spectrum from the APT analysis capturing detected Pt species, while (b) displays another portion of the mass spectrum capturing various electrolyte species such as Li, C and H. (c) Shows a 3D reconstruction of the created needle specimen with (d) displays 2D contour plots showing the relative decomposed concentration of electrolyte, Pt and Cr species.

The APT analysis generated a mass spectrum and detection hit-map which can be found in detail in Fig. S1. Two specific ranges have been selected based on peaks containing species of interest that would be expected from the electrode and electrolyte and are shown in Fig. 8(a) and (b). The electrolyte used for this experiment was LiPF_6_ in EC/DMC (C_3_H_4_O_3_/OC(OCH_3_)_2_), meaning species containing variations of Li, P, F, O, C and H were assigned as being part of the electrolyte. While residual H will always be present within the atom probe chamber and can obscure H atoms that have been detected from the created specimen,^67^ it remains challenging to unambiguously distinguish between H originating from the specimen and that from the vacuum environment. For this work all detected H species have been assigned as “electrolyte species” in the analysis. From Fig. 8(a) it can be seen that Pt could be readily identified, along with peaks pertaining to PtH and in (b) various species from the electrolyte can be identified such as Li, C and H.

Through the combination of the generated mass spectrum and detection hit-map, a 3D reconstruction was generated giving a clear picture of the positions of the detected ions within the created needle specimen and is shown in Fig. 8(c). To give a clearer picture of the ions within the 3D reconstruction 2D contour plots showing the relative concentration of decomposed electrolyte species, Pt species and Cr species is shown in Fig. 8(d). Decomposed 2D contour plots of Pt, Cr and all electrolyte species in *X*–*Z* and *Y*–*Z* planes can be found in Fig. S2 and S3. Based on the reconstruction and 2D contour plots it is evident three distinct regions have been captured. On the bottom a large concentration of Cr has been detected, translating to the Cr “fill in” from the sample preparation process. Following this there is a large concentration of Pt species, capturing the Pt electrode from the nanochip and above this there is a large concentration of “electrolyte species” from the electrolyte and a smaller concentration of Cr from the Cr coating process at the end of the sample preparation. These distinct regions showcase that the interface between the electrolyte and electrode from the LCTEM has been captured to some extent. The 3D reconstruction of the region containing the electrolyte species appears to have evaporated non uniformly, with an observable heterogenous distribution of Li suggesting possible Li ion migration during analysis. While a Cr coating was applied and can be seen in the 2D contour plot, it was not sufficient to stop Li migration during analysis. Details on all electrolyte and Cr detected species are represented in Fig. S4 and S5 as bar charts displaying atom type *versus* total count.

## Conclusion and outlook

This work provides a novel and reproducible workflow for site-specific cryogenic APT sample preparation of liquid–solid interfaces from MEMs based *in situ* nanocells. Through the integration of cryogenic inert gas transfer, a cryogenic PFIB/SEM and an inert nitrogen glovebox with LN_2_ capabilities, we have successfully preserved, extracted and analysed a Li electrolyte–Pt electrode interface from an *in situ* LCTEM electrochemical nanocell using cryogenic APT, maintaining the sample in its state of interest throughout the entire process. The developed workflow overcomes the limitations of traditional LCTEM by enabling sub nanometre three-dimensional compositional analysis, complementing the *operando* capabilities and dynamic nanoscale imaging offered by LCTEM.

Our findings demonstrate the feasibility of using cryogenic APT in combination with other microscopy techniques, to take advantage of the critical chemical and compositional information APT can offer at (near-)atomic length scales, which is not accessible due to resolution constraints in many liquid-based microscopy techniques. This cryogenic workflow can be adapted to a wide range of liquid based *in situ* MEMs studies including various battery materials, catalytic reactions, and corrosion studies across a wide range of techniques such as LCTEM, liquid cell atomic force microscopy, liquid cell synchrotron X-ray imaging *etc.*

Challenges remain for analysing frozen liquid–solid interfaces using cryo APT, demonstrated by the potential Li migration effects within the data shown. Further research is required to fully understand and minimise any such effects to provide fully representative analysis. This workflow opens up the field of correlative *operando*/*in situ* microscopy and cryogenic techniques. The integration of cryogenic multimodal approaches, including cryogenic electron microscopy and cryogenic APT, has the potential to revolutionise our understanding of solid–liquid interactions at the nanoscale.

## Author contributions

M. C. conceived the idea of combining liquid cell TEM with site specific cryo APT analysis. N. M., J. O. D., S. R. J., L. W., M. S. C. conducted the SEM and FIB experiments, N. M., and M. S. C. did the *in situ* TEM experiments, N. M., J. O. D., B. G., M. S. C., analysed the APT specimens and processed the data. N. M. and M. S. C. lead the publication writing. All authors discussed the results and contributed to the final version of the manuscript. The project was supervised by B. G., M. P. R. and M. C.

## Conflicts of interest

The authors declare that they have no known competing financial interests or personal relationships that could have appeared to influence the work reported in this paper.

## Supplementary Material

NH-010-D5NH00310E-s001

## Data Availability

The data supporting this article have been included as part of the main article and SI. See DOI: https://doi.org/10.1039/d5nh00310e.
