# Supplementary material for: A workflow for correlative in situ nanochip liquid cell transmission electron microscopy and atom probe tomography enabled by cryogenic plasma focused ion beam
Source: Nanoscale Horiz. 2025 Sep 15;10(12):3486–98. doi: 10.1039/d5nh00310e (PMC12516483; doi:10.1039/d5nh00310e)
Supplement: NH-010-D5NH00310E-s001 [file NH-010-D5NH00310E-s001.pdf]

Supplementary Information (SI) for Nanoscale Horizons.
This journal is © The Royal Society of Chemistry 2025

Supporting information

A Workflow for Correlative In-situ Nanochip Liquid Cell Transmission Electron Microscopy and Atom Probe Tomography Enabled by Cryogenic Plasma Focused Ion Beam

Neil Mulcahy[1], James O. Douglas[1], Syeda Ramin Jannat[1], Lukas Worch[1], Geri Topore[1], Baptiste Gault[1,2], Mary P. Ryan[1], Michele Shelly Conroy[1*]

1. Department of Materials and London Centre for Nanotechnology, Imperial College London, Exhibition Road, London SW7 2AZ, U.K.

2. Max Planck Institute for Sustainable Materials, Max-Planck-Str. 1, 40237 Düsseldorf, Germany

*Corresponding author: mconroy@imperial.ac.uk

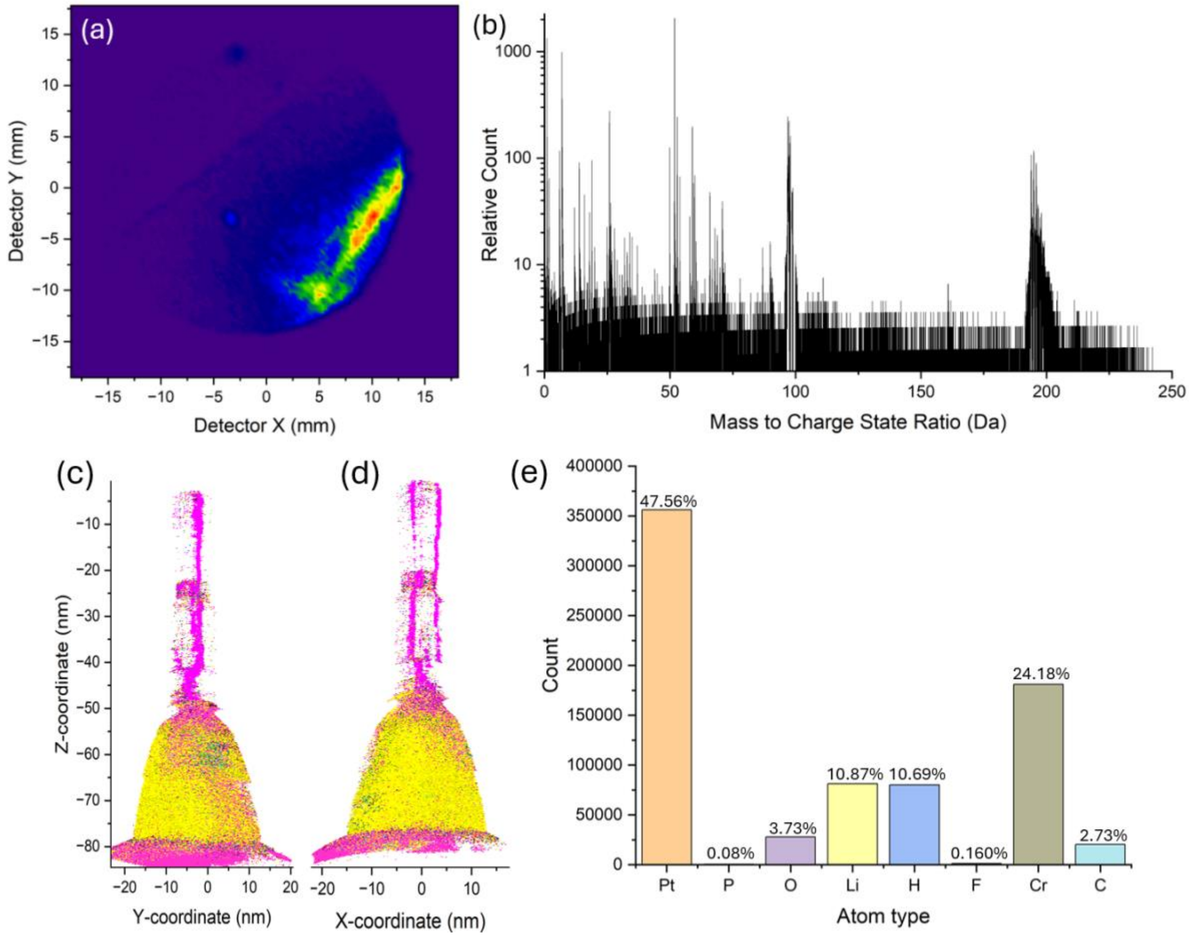

Fig. S1: (a) Detection hit map and (b) full mass spectrum from APT analysis of Pt-Li electrolyte interface. (c) 3D reconstructions generated from (a) and (b) in X and Y directions. (e) a bar chart showing total decomposed species count versus atom type, with the percentage of each type of atom present from all the ranged species listed.

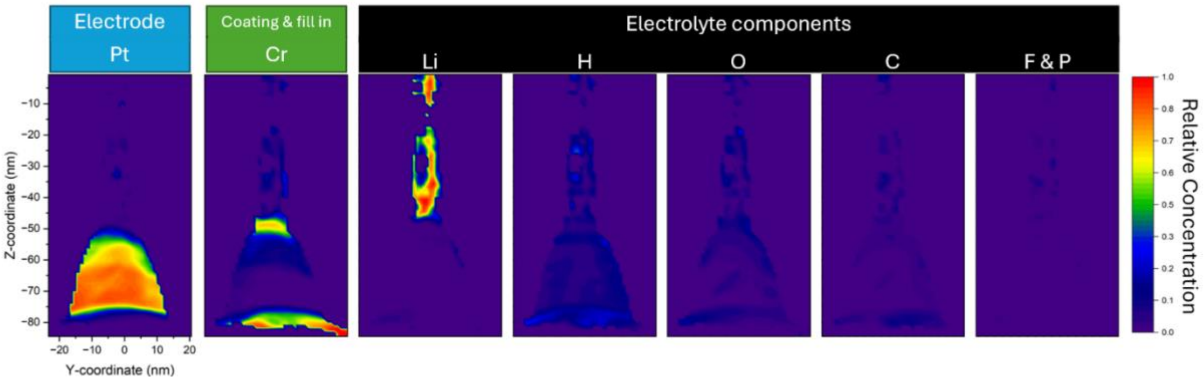

Fig. S2: 2D contour plots showing the relative concentrations of Pt, Cr and various electrolyte species including Li, H, O, C , F and P within the 3D reconstruction in the Y-Z plane.

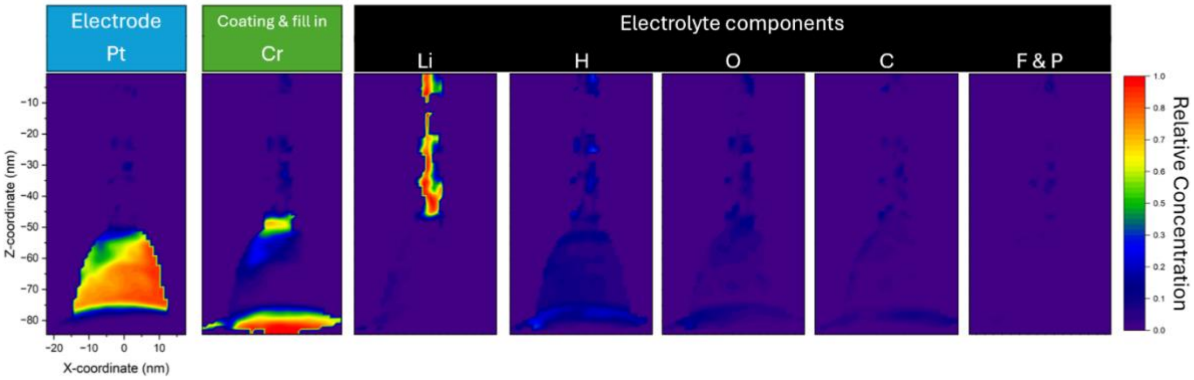

Fig. S3: 2D contour plots showing the relative concentrations of Pt, Cr and various electrolyte species including Li, H, O, C , F and P within the 3D reconstruction in the X-Z plane.

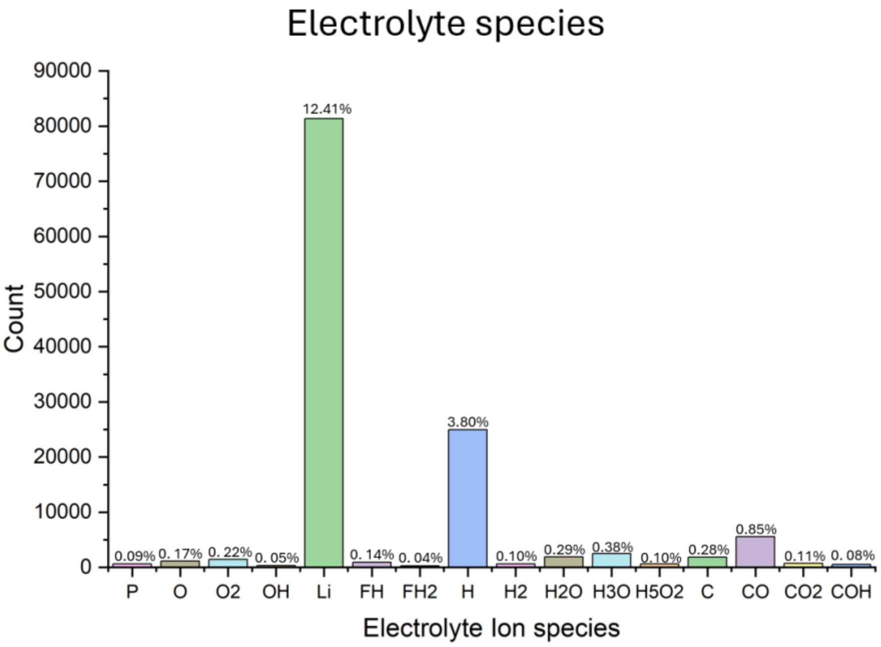

Fig. S4: Bar chart showing every ionic species that contained electrolyte species (H,O,P,F,Li) versus detected count. The percentage of detected species versus total count of all detected species is shown over each bar.

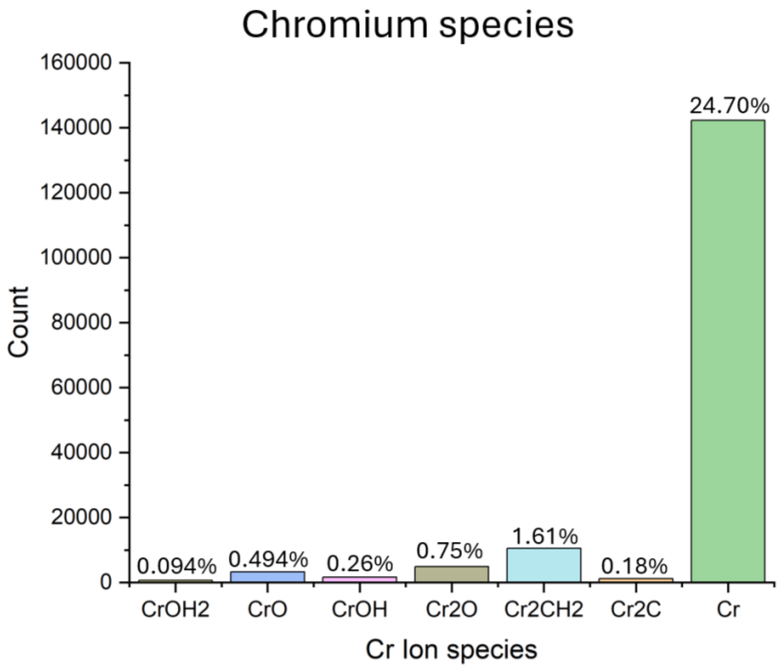

Fig. S5: Bar chart showing every ionic species that contained Cr versus detected count. The percentage of detected species versus total count of all detected species is shown over each bar.
